# Antimutagenicity and Antiproliferative Studies of Lipidic Extracts from White Shrimp (*Litopenaeus vannamei*)

**DOI:** 10.3390/md8112795

**Published:** 2010-11-08

**Authors:** Griselda Wilson-Sanchez, Carolina Moreno-Félix, Carlos Velazquez, Maribel Plascencia-Jatomea, Anita Acosta, Lorena Machi-Lara, María-Lourdes Aldana-Madrid, Josafat-Marina Ezquerra-Brauer, Ramón Robles-Zepeda, Armando Burgos-Hernandez

**Affiliations:** 1 Departamento de Investigación y Posgrado en Alimentos, Universidad de Sonora, Apartado Postal 1658, Hermosillo, Sonora, Mexico; E-Mails: gris_wilson@hotmail.com (G.W.-S.); carolina_2400@hotmail.com (C.M.-F.); mplascencia@guayacan.uson.mx (M.P.-J.); laldana@guayacan.uson.mx (M.-L.A.-M.); ezquerra@guayacan.uson.mx (J.-M.E.-B.); 2 Departamento de Ciencias Químico Biológicas, Universidad de Sonora, Apartado Postal 1658, Hermosillo, Sonora, Mexico; E-Mails: velaz@guayacan.uson.mx (C.V.); anitacheecks@hotmail.com (A.A); rrobles@guayacan.uson.mx (R.R.-Z.); 3 Departamento de Investigación en Polímeros y Materiales, Universidad de Sonora, Apartado Postal 1658, Hermosillo, Sonora, Mexico; E-Mail: lmachi@polimeros.uson.mx (L.M.-L.)

**Keywords:** antimutagenicity, antiproliferation, cultured shrimp

## Abstract

An organic extract from fresh shrimp (*Litopenaeus vannamei*) was studied for antimutagenic and antiproliferative properties using *Salmonella typhimurium* tester strains TA98 and TA100 with metabolic activation (S9) and a cancer cell line (B-cell lymphoma), respectively. Shrimp extract was sequentially fractionated by thin layer chromatography (TLC) and each fraction was tested for antimutagenic and antiproliferative activities. Crude organic extracts obtained from shrimp reduced the number of revertants caused by aflatoxina B_1_, showing a dose-response type of relationship. Sequential TLC fractionation of the active extracts produced several antimutagenic and/or antiproliferative fractions. These results suggested that the lipid fraction of the tested species contained compounds with chemoprotective properties that reduce the mutagenicity of AFB_1_ and proliferation of a cancer cell line.

## 1. Introduction

During the last two decades several kinds of chemical mutagens and carcinogens have been found to be present in food. Some of them are food contaminants such as mycotoxins, pesticides, *etc.*, and some others are present in food as a consequence of the processes applied to them (e.g., pyrolysates, nitrosamines, policyclic aromatic hydrocarbons, *etc.*) [1]. However, in our diet, a great variety of compounds have been found to have properties that are beneficial for the consumer. Among the types of “functional” compounds found in food are anti-cholesterolemic compounds, antioxidants, antiviral, *etc.*, including antimutagenic and antiproliferative agents. These compounds include thiocyanates, indole-3-carbinol, allyium compounds, linoleic acid (also found as conjugated linoleic acid), polyunsaturated fatty acids, caffeic acid phenethyl ester (CAPE) and flavonoids such as pinocembrin, rutin, naringenin, and hesperetin, respectively [2].

Omega-3 and omega-6 fatty acids are components of lipid fraction of fish that have been associated with the prevention of cardiovascular diseases and cancer [3–5]. These fatty acids are structurally similar to linoleic and linolenic acids, which have been shown to be antimutagenic and anticarcinongenic [3,6,7]. Based on these facts, this research work attempted to detect the presence of antimutagenic and antiproliferative compounds in shrimp, a highly consumed seafood, worldwide.

## 2. Results and Discussion

### 2.1. Antimutagenicity

**Figure 1:**
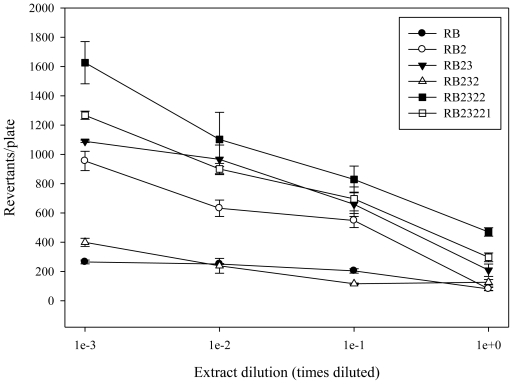
Antimutagenic potential of most active fractions sequentially isolated from white shrimp. *Salmonella* test strain TA98 was exposed to different concentrations of the fractions obtained throughout the fractionation procedure. All values represent mean of triplicate determinations ± standard deviation.

**Figure 2:**
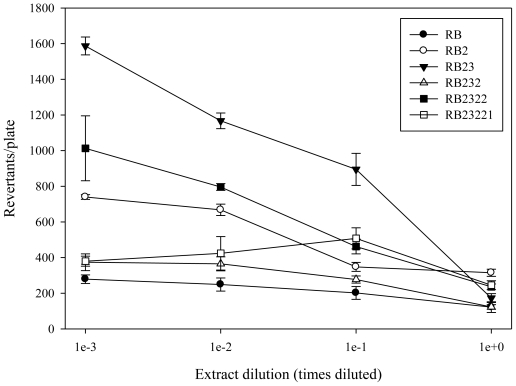
Antimutagenic potential of most active fractions sequentially isolated from white shrimp. *Salmonella* test strain TA100 was exposed to different concentrations of the fractions obtained throughout the fractionation procedure. All values represent mean of triplicate determinations ± standard deviation.

**Scheme 1:**
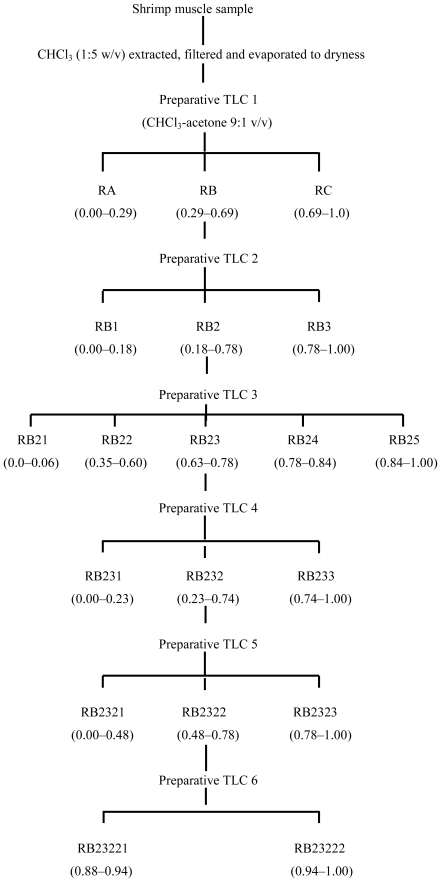
Schematic for separation and isolation of antimutagenic fractions from shrimp. Numbers in parentheses are R_f_ values.

Shrimp tail muscle was extracted and fractionated using TLC, and the fractions obtained (Scheme 1) were tested for antimutagenicity. The antimutagenic potential of the fractions that were selected for further fractionation are shown in Figures 1 and 2. After first TLC procedure (TLC 1) fractions RA, RB, and RC were obtained. Antimutagenicity testing showed all extracts to have an inhibitory effect on the mutagenicity of 500 ng of AFB_1_ for both tester strains suggesting the presence of antimutagenic compounds in all of them. Fraction B was selected for further fractionation, as it showed a well defined dose-response type of relationship. After second TLC isolation procedure, three fractions were obtained from RB (Scheme 1). Fraction RB2 (R_f_ = 0.18–0.78) decreased the reversion rate achieved by 500 ng AFB_1_ suggesting that antimutagenic compounds were contained in this fraction. Although other fractions also decreased reversion caused by AFB_1_, dose-response type of relationships were not as consistent in both tested strains as that observed for RB2.

Therefore, RB2 was selected for further fractionation. After the third TLC isolation procedure, five fractions were obtained from RB2 (Scheme 1). Antimutagenic testing performed on these fractions showed that fractions RB23 differed from the rest having effectively inhibited the mutagenicity of AFB_1_ induced in both tester strains. RB23 was selected for further fractionation. Fractionation of RB23 (TLC 4) resulted in three regions, RB231, RB232, and RB233. Antimutagenesis testing revealed that fraction RB232 more efficiently decreased tester strains reversion rate than RB231 and RB233, showing a dose-response relationship with a lower slope. Therefore, RB232 was selected and fractionated by means of a TLC 5 procedure. TLC 5 developed in only two fractions that were named RB2321 and RB2322. The antimutagenicity assay performed, showed RB2322 as the fraction that inhibited the mutagenicity of AFB_1_ in both tester strains, and its contents were fractionated using a sixth TLC procedure. TLC 6 applied to RB2322 resulted in only two bands, RB23221 and RB23222. The contents from these bands were extracted and tested for antimutagenicity. Results from this assay showed that both bands contained compounds that inhibited the mutagenicity of AFB_1_ in a dose-response type of relationship. The contents from both bands were subjected to further TLC procedures using a different solvent system without successful fractionation.

### 2.2. Antiproliferative activity

**Scheme 2:**
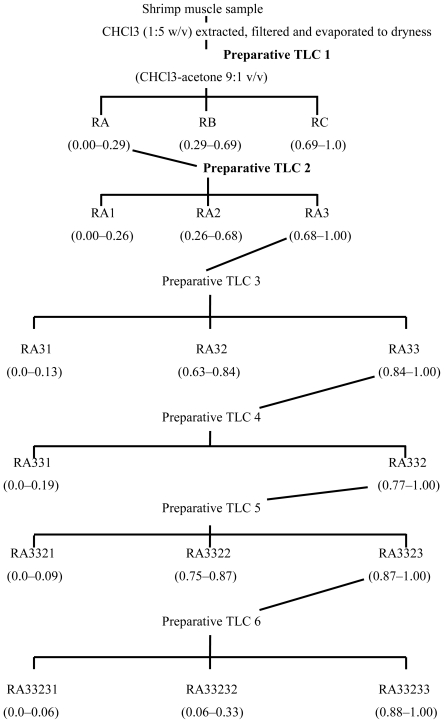
Schematic for separation and isolation of antiproliferative fractions from shrimp. Numbers in parentheses are R_f_ values.

In order to investigate the presence of antiproliferative agents in shrimp lipidic extract, a fractionation procedure parallel to that carried out for antimutagens was performed (Scheme 2).

**Figure 3:**
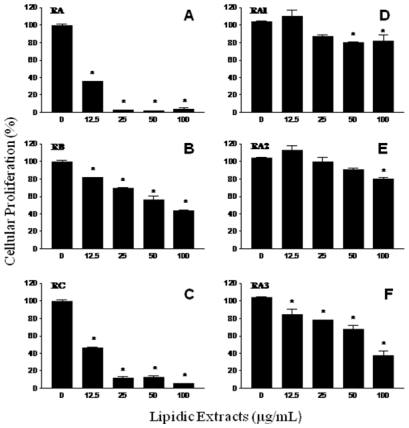
Antiproliferative effect of lipidic extracts from white shrimp on murine cancerous cell lines. Murine cancer M12.C3.F6 cell lines were treated with different dose lipidic extracts during 48 h. Cellular proliferation was determined by standard MTT assay (3-(4,5-dimethylthiazol-2-yl)-2,5-diphenyltetrazolium bromide). The results shown are representative from at least three independent experiments. All values represent mean of triplicate determinations ± standard deviation. Significant differences (P < 0.05) from control cell cultures are marked with an asterisk. Control cell cultures were incubated with DMSO (0.5%).

The fractions RA, RB and RC obtained from the first TLC fractionation were tested on the antiproliferative assays. All shrimp fractions showed antiproliferative activity on the murine cancer cell line M12.C3.F6 (B-cell lymphoma) in a concentration-dependent manner (Figure 3A–C). However, only fractions RA and RC were able to inhibit the cellular proliferation beyond 50%, at the lowest doses tested (12.5 and 25 μg/mL). The highest level of cellular proliferation inhibition was observed for fraction RA (above 95% for the second lowest dose tested (25 μg/mL); therefore, this shrimp fraction was selected for further fractionation).

**Figure 4:**
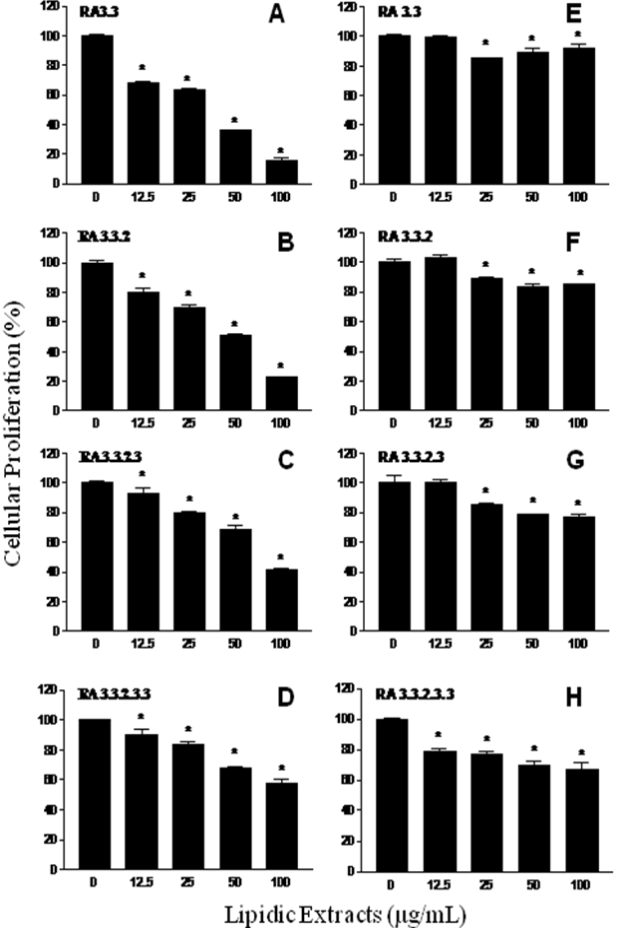
Antiproliferative effect of lipidic extracts from white shrimp on murine cancer and non-cancerous cell lines. Murine cancer M12.C3.F6 (A, B, C y D) and non cancerous L-929 (E, F, G y H) cell lines were treated with different dose lipidic extracts during 48 h. Cellular proliferation was determined by standard MTT assay (3-(4,5-dimethylthiazol-2-yl)-2,5-diphenyltetrazolium bromide). The results shown are representative from at least three independent experiments. All values represent mean of triplicate determinations ± standard deviation. Significant differences (P < 0.05) from control cell cultures are marked with asterisk. Control cell cultures were incubated with DMSO (0.5%).

From the second TLC fractionation step, fractions RA1 (R_f_ = 0.00–0.26), RA2 (R_f_ = 0.26–0.68), and RA3 (R_f_ = 0.68–1.0) were obtained (Scheme 2) and tested for antiproliferation activity (Figure 3D–F). Fractions RA1 and RA2 were able to inhibit cellular proliferation up to 20% at the highest dose tested (100 μg/mL). However, a 60% cellular proliferation inhibition was observed in cancer cell cultures exposed to fraction RA3; therefore, this fraction was selected to continue with the fractionation process. Figure 4A–D shows the antiproliferative activities of shrimp fractions obtained from subsequent TLC fractionation (TLC 3, TLC 4, and TLC 5) of fraction RA3. All fractions derived from RA3 had significant inhibitory effect on the growth of the cancer cell line M12.C3.F6 tested. In contrast, RA3-derived fractions showed a low antiproliferative effect on the murine non-cancerous cell line L-929 (Figure 4E–H).

In previous studies performed in our laboratory, we have detected the presence of antimutagenic compounds in yellowtail fish (*Seriola lalandi*), lisa fish (*Mugil cephalus*), and cazon fish (*Mustelus lunulatus*) [8]. Chemical components of marine organisms have been associated to a variety of chemopreventive properties. Omega-3 polyunsaturated fatty acids (n-3PUFAs) have been implicated in chemoprevention of cancer [5,9,10]. Omega-3 PUFAs have been associated with cancerous tumor growth suppression [11,12] and reduced risk of prostate cancer [13,14].

Therefore, the current research focused on the isolation of compound(s) present in a lipidic extract from shrimp that would cause an inhibition of the mutagenicity of AFB_1_ or cellular proliferation of cancer cell lines, or both. All fractions obtained after the first fractionation step decreased the number of revertants per plate in a dose-response type of relationship in both tester strains, suggesting the presence of antimutagens in every fraction. All of the fractions were able to cause an inhibition of AFB_1_ mutagenicity close to 50%, even at the highest dilution tested. At this point, we could have selected any of the fractions to continue with the isolation process. However, for both strains, the lower number of revertants/plate was observed for fraction RB, suggesting that contents from this fraction might have a higher potency in inhibiting the mutagenicity of AFB_1_; therefore, RB was selected for further fractionation. Additional studies on the antimutagenic properties of fractions RA and RC are recommended.

Fraction RB was fractionated into another three groups of bands coded as RB1, RB2, and RB3 (Scheme 1). Antimutagenesis testing performed on the extracts from these fractions showed that fraction RB2 (Figures 1 and 2) had the most consistent dose-response type of relationship in both tester strains. Fractions such as RB1 and RB3 partially inhibited the mutagenicity of AFB_1_ towards both tester strains; however, they fail to show a dose-response type of relationship. However, these results may suggest the presence of antimutagenic compounds which are minor components of fraction RB or have less antimutagenic potency than those contained in fractions RB2. Based on the dose-response relationship and antimutagenic potency, fractions RB2 was selected for further fractionation.

Fraction RB2 was separated into five bands which were also tested for antimutagenicity. From the five extracts obtained and tested, fractions RB23 caused the highest inhibition of AFB_1_ mutagencity (more than 50% AFB_1_ inhibition was achieved with undiluted RB23 for both tester strains) showing a dose-response type of relationship. Fractions RB22, RB24, and RB25 showed antimutagenicity when undiluted; however, this activity was lost upon first dilution. A possible reason for this apparent loss of antimutagenicity after the third step in the fractionation process is the possible existence of synergistic antimutagenic compounds that may loss their antimutagenic properties when separated. However, the possible loss of material throughout the fractionation process is not discounted. Based on the above, the fractionation of RB23 proceeded. The contents of RB23 were fractionated into three regions on the TLC plate: RB231 (R_f_ = 0.00–0.18), RB232 (R_f_ = 0.18–0.78), and RB233 (R_f_ = 0.78–1.0). Results from the antimutagenicity assay showed that RB232 inhibited AFB_1_ to a greater extent than the other two fractions. These results suggested that antimutagenic compounds were concentrated in this fraction located in the middle portion of the TLC plate. The contents of this fraction were extracted and subjected to a fifth TLC procedure in order to continue with the isolation of antimutagenic compounds from shrimp. TLC 5, RB232 was then divided into two fractions, RB2321 and RB2322. Although both fractions showed antimutagenic potential, contents from RB2322 inhibited AFB_1_ mutagenicity in both tester strains showing a consistent dose-response type of relationship. The sixth TLC procedure applied to RB2322 resulted in another two fractions, RB23221 and RB23222. Contents from both fractions were localized in the upper portion of the plate, showing their affinity for the solvent used (CHCl_3_-acetone 9:1). Both were antimutagenic, showing similar dose-response relationships, which suggest the presence of at least two compounds with similar antimutagenic characteristics. Attempts to fraction both, RB23221 and RB23222 using different solvents system were unsuccessful, suggesting that the chemical nature of the compounds contained in both fractions are similar. Further studies on the chemical/structural characterization of RB23221 and RB23222 are under way.

As mentioned before, three regions (RA, RB, and RC) were obtained from the fractionation of the lipidic extract from shrimp. Fraction RB was the most antimutagenic; however, all fractions showed antiproliferative activity, with RA and RC being the fractions that had the highest inhibition of cancer cell proliferation (Figure 3). These results suggest that all three fractions contained both, antimutagens and antiproliferative compounds; however, this investigation focused on the search for the compounds with the most potent activities. Fractions RA and RC inhibited murine cancer cell proliferation in a dose-dependent manner; however, at the lowest dose tested (12.5 μg/mL), fraction RA inhibited more than 50% of cell proliferation compared to the control. Therefore, RA was considered the most potent antiproliferative fraction and was selected for further fractionation.

Of the three fractions derived from fraction RA (RA1, RA2, and RA3), neither RA1 nor RA2 were able to significantly inhibit murine cancerous cell proliferation. However, fraction RA3 at a dose of 100 μg/mL inhibited more than 60% of cell proliferation compared to the control (Figure 3F). These results suggest that antiproliferative compound(s) of fraction RA were partitioned to fraction RA3, therefore this was subjected to additional TLC procedures.

From the third TLC fractionation step (fraction RA3), three fractions (RA31, RA32, and RA33) were obtained, of which fraction RA33 showed the highest inhibition of cancer cell proliferation (close to 90%). This might suggest that components with this type of biological activity were isolated in this fraction and possibly separated from other interfering compounds. However, further studies are needed in order to characterize these active fractions.

Lipidic fractions from white shrimp substantially delayed the growth of the murine cancer cell M12.C3.F6, but the growth of normal murine cell line L-929 was considerably less affected. These observations suggest that active constituents of white shrimp have a preferential antiproliferative effect on cancer cell lines.

### 2.3. Partial chemical/structural characterization studies

To investigate the presence of functional groups and structural features, that may contribute to elucidate the chemical/structural nature of the bioactive compounds present in both antimutagenic and antiproliferative fractions from shrimp, FT-IR and ^1^H NMR studies were carried out. When bioactive fractions were analyzed by ^1^H NMR, the spectra obtained showed broad peaks located in the 1.3 ppm region; these signals are attributed to the presence of methylenic protons from long chain fatty acids, such as those present in ω-3 fatty acids. Additional contributions from lipid resonances (0.9–1.7 ppm region) are also present, where the wide peak at 0.9 ppm region is associated to methyl groups [17]. FT-IR spectroscopy performed on bioactive fractions from shrimp resulted in spectra that coincided in a typical signal for carbonyl function group; the peaks located in the 1600–1680 range could be related to the unsaturated hydrocarbons featuring C=C, with attached hydrogens. The C-H stretch vibrations at 2900 cm^−1^ can be attributed to methylenic groups; this is in agreement with results from NMR which suggest the presence of fatty acids. In addition, the spectrum of both the antimutagenic fraction (RB23221) and the antiproliferative fraction (RA33233), showed a typical signal of hydroxyl group above 3000 cm^−1^; this is also in accordance with a fatty acid structure.

## 3. Experimental Section

### 3.1. Testing species

Shrimp (*Litopenaeus vannamei*) was obtained from Bahía de Kino, Sonora, México (approximately 2000 km northwest México City, México) and transported in ice to the University of Sonora Seafood Laboratory. Edible portions of shrimp were separated, fresh-packed, and stored at −25 °C until needed for further analysis.

#### Shrimp extract

A 100 g portion of shrimp muscle and five parts of CHCl_3_ were homogenized in a blender at high speed for 1 min. Resulting mix was poured into a flask and agitated during 30 min with the aid of a Wrist Action Burrel Shaker (Burrel Corporation, Pittsburg, PA, U.S.). The mix was filtered through Whatman No. 4 filter paper with vacuum and the filtrate was evaporated to dryness under N2 stream.

### 3.2. Fractionation of shrimp extract

The fractionation of shrimp extract was performed according to Burgos-Hernández *et al.* [8]. A 2.0 mL aliquot of shrimp muscle extract was applied onto a 1.0 mm thick silica gel coated preparative TLC plate and developed with chloroform-acetone (9:1 v/v). Fluorescent bands, identified with their Rf, were scraped off the plate and the silica gel was extracted with 2 × 25 mL chloroform-methanol-acetone (9:1:1 v/v/v). Extracts were re-suspended and serially diluted in DMSO and tested for antimutagenicity and antiproliferation. When either antimutagenic or antiproliferative bands were detected, their contents were obtained again from a fresh muscle sample following the same procedure and were subjected to further fractionation (Schemes 1 and 2).

### 3.3. Bacterial cultures

*Salmonella typhimurium* TA98 and TA100 were kindly provided by Dr. B. N. Ames, Department of Biochemistry (University of California at Berkeley, CA, U.S.). Fresh overnight tester strain cultures, to which DMSO was added as cryoprotective agent, were stored at −80 °C. Tester strains were checked routinely to confirm genetic features using the procedure described by Maron and Ames [15]. Metabolic activation system S9 mix (Aroclor 1254-induced, Sprague-Dawley male rat liver in 0.154 M KCl solution) was purchased from Molecular Toxicology, Inc. (Annapolis, MD, U.S.) and stored at −80 °C.

### 3.4. Antimutagenicity test

Dry extracts, obtained either directly from shrimp or from preparative-TLC fractionation of extracts, were reconstituted and serially diluted with DMSO, and were spiked with pure AFB_1_ (Sigma-Aldrich, St. Louis, MO, U.S.) to a final concentration of 500 ng of AFB_1_/100 μL. Residual mutagenicity of AFB_1_ was assayed using the standard plate incorporation procedure described by Maron and Ames [15]. Different AFB_1_ concentrations were used as a control for both tester strains. All assays were performed in triplicate.

### 3.5. Cell lines

Cell lines NCTC clone L929 (normal subcutaneous connective tissue) was purchased from the American Type Culture Collection (ATCC, Rockville, MD, U.S.). The M12.C3.F6 (murine B-cell lymphoma) cell line was kindly provided by Dr. Emil R. Unanue (Department of Pathology and Immunology, Washington University in St. Louis, MO, U.S.). All cell cultures were cultured in Dulbecco’s modified Eagle’s medium (DMEM) supplemented with 5% heat inactivated fetal calf serum and grown at 37 °C in an atmosphere of 5% CO2.

### 3.6. Antiproliferation assay

To evaluate the effect of shrimp crude extracts and their fractions on the proliferation of different cancer cell lines, cell proliferation was determined using the standard MTT assay (3-(4,5-dimethylthiazol-2-yl)-2,5-diphenyltetrazolium bromide) [16]. Briefly, 10,000 cells (50 μL) were plated in each well of a flat 96 well plate. After 12 h incubation at 37 °C in an atmosphere of 5% CO2 to allow cell attachment, the cell cultures were incubated with 50 μL of medium containing various concentrations of either crude extract or fraction, and the cell cultures were incubated for 48 h. The crude extract or fraction was first pre-suspended in DMSO and then diluted in DMEM media. Control cell cultures were incubated with DMSO (final concentrations of DMSO 0.06–0.5%). Control cell cultures did not show any evidence of cell damage. In the last 4 h of the cell culture, 10 μL of MTT stock solution (5 mg/mL) were added to each well [16]. Formazan crystals formed were dissolved with acidic isopropanol, and the plates were read in an ELISA plate reader (Benchmark Microplate Reader, Bio-Rad, Hercules, CA, U.S.), using a test wavelength of 570 nm and a reference wavelength of 630 nm. Plates were normally read within 15 minutes of adding isopropanol. Data were analyzed using analysis of variance (ANOVA) with Tukey-Kramer and Duncan’s multiple comparison tests (Number Cruncher Statistical Software (NCSS 2000).

### 3.7. Partial chemical/structural characterization studies

To obtain information that may contribute to a partial chemical/structural characterization of the compounds contained in the bioactive fractions, Fourier Transform Infrared (FT-IR) and nuclear magnetic resonance (^1^H NMR) studies were performed. For FT-IR spectroscopy, thin films were formed with samples from the bioactive fractions on ZnSe cells and spectra were obtained using a GX Perkin Elmer equipment. To obtain the nuclear magnetic resonance spectra, bioactive fractions were dissolved in CDCl_3_ and analyzed in a Bruker ADVANCE 400 spectrometer using tetramethylsilane (TMS) as a reference.

## 4. Conclusions

Additional studies need to be conducted to define and characterize, at the chemical and biochemical level, the preferential effect of shrimp fractions on murine cancer cell lines. Results from this study suggest that there are various groups of compounds in the lipid fraction of shrimp that are biologically active against either the mutagenicity of AFB_1_ or murine cancerous cell proliferation, or against both. Although n-3PUFAs may be considered as factors partially responsible for the antimutagenicity observed in the first fractions obtained in this study, the isolation and identification of the actual antimutagens in fish is the focus of our ongoing research.

## Figures and Tables

**Figure 5:**
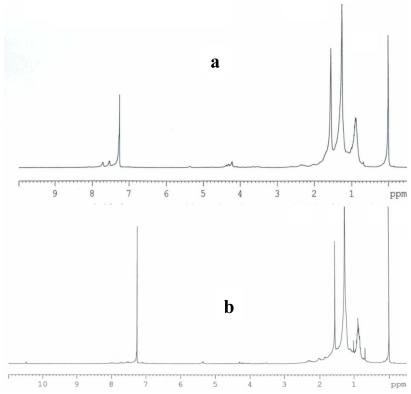
^1^H NMR spectra (CDCl_3_/TMS) of antimutagenic fraction RB23221 (a), and antiproliferative fraction RA33233 (b); both obtained from a lipidic extract from white shrimp.

**Figure 6:**
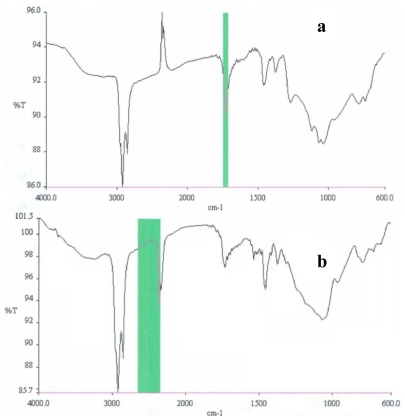
FT-IR spectra of antimutagenic fraction RB23221 (a), and antiproliferative fraction RA33233 (b), both obtained from a lipidic extract from white shrimp.
